# Indents

**Published:** 1919-05

**Authors:** 


### INDENTS

**☞Brief Articles of Technical or Scientific Value will receive notice and comment. Contributions invited.**

---

CHARCOAL AND ULCERATIVE STOMATITIS. Although ulcerative stomatitis has come into prominence as a war disease, and its wide prevalence and virulence was unquestionably due to war conditions, the dental surgeon's interest in this disorder should not by any means slacken or fall away on the cessation of hostilities. Ulcerative stomatitis is an infective disease, and in many treated cases, where it has been checked and the mouth restored to apparent health, there is the liability of recurrence. And in an instructive paper on "Some Methods of Treatment of Ulcerative Stomatitis" (*British Dental Journal*, March 15, 1919), we are reminded by Mr. J. H. Hall that an epidemic is not a remote possibility, especially in view of the "depressing and devitalising influences of mental strain," from which so many of our people have not yet been able to emerge. Useful and suggestive practical hints for post-war service may be gathered from the methods of treatment described by Mr. Hall. The use of an oxygen-carrying substance is very helpful in checking the growth of the anacrobic micro-organisms, and with that object Mr. Hall—with no faith in the value of hydrogen proxide—employs powdered wood charcoal. His reference to the use of charcoal may be quoted here, as it is a simple and valuable adjuvant in the full system of treatment: "The patient is instructed to take a teaspoonful of charcoal last thing at night on retiring, and to retain as much of it as possible in the mouth until morning. To accomplish this it should be taken when the patient is undressed and ready to get into bed. On rising in the morning the teeth and gums will be black with the charcoal, but a single rinsing with water will remove every trace. Charcoal used thus completely destroys the horrible odor which is so characteristic of ulcerative stomatitis. It cleans the teeth and gums, so that in those severe cases exhibiting excessive gingival hypertrophy with haemorrhage where the use of a tooth brush is for the time impossible, the charcoal powder prevents the mouth from getting foul. It prevents extension of the disease by contact. It is during the hours of sleep when the oral functions are in abeyance and the lips and cheeks in prolonged contact with gums and teeth that extension takes place. Coat the surfaces with charcoal and infection from contact is almost impossible. The charcoal also cleanses the ulcerated patches, absorbs and disinfects the ulcerous exudate, and when rinsed out brings away the accumulated sloughs."—*Dental Record.*

---

DENTAL GRANULOMA CAUSE OF HICCOUGH. Dr. M. W. Hoolar, writing to *Oral Hygiene*, tells of a patient who was suffering with a sore right upper cuspid, the peridontal structures indicating pyorrhea, and with symptoms of an alveolar abscess. He had also been afflicted for three days with uncontrollable hiccoughs. The tooth was extracted and a large-sized granuloma was attached to the apical end of the root. The hiccoughs stopped immediately after the extraction and have not reappeared since. Was there a reflex irritation from the granuloma or apical region sufficient to induce the muscular reactions of hiccough?

---

FLASKING. Before pouring the plaster of Paris into the flask to form the mould, thoroughly dust the inside of the flask with French chalk to prevent the plaster sticking to it. The vulcanized case can then be quite easily removed without bruising the flask.—*Oral Health.*

---

SOLDER FOR ALUMINUM PLATE. A French patent has been issued for an aluminum solder, which consists of Al. 95 parts, copper 2 parts, antimony, bismuth and zinc, each one part. The aluminum must be protected by a flux; viz., a thin layer of phosphoric acid.—Translation by H. Prinz.

---

TO DRILL CAVITIES IN PORCELAIN TEETH. A diamond drill of good quality is not easy to obtain and requires great care in its use to prevent destruction. To cut cavities without a diamond drill I would suggest the use of a paste made from corundum powder, turpentine and camphor. After the pit has been cut with a barrel-shaped miller stone, fill it with some of this paste and with a copper mandrel of the desired size, drill two retaining points.—F. W. Frahm, in *Pacific Gazette.*

